# Cooling of a vial in a snapfreezing device without using sacrificial cryogens

**DOI:** 10.1038/s41598-019-40115-6

**Published:** 2019-03-05

**Authors:** Michiel A. J. van Limbeek, Sahil Jagga, Harry Holland, Koen Ledeboer, Marcel ter Brake, Srinivas Vanapalli

**Affiliations:** 1 Energy, Materials and Systems Group, University of Twente, 7500 AE Enschede, The Netherlands; 2 Physics of Fluids Group, MESA+ Institute, University of Twente, 7500 AE Enschede, The Netherlands

## Abstract

A fresh and frozen high-quality patient bio-sample is required in molecular medicine for the identification of disease-associated mechanism at molecular levels. A common cooling procedure is immersing the tissue enclosed in a vial in a coolant such as liquid nitrogen. This procedure is not user friendly and is laborious as reducing the lag time from excision time to freezing depends on the logistic organizational structure within a hospital. Moreover snapfreezing must be done as soon as possible after tissue excision to preserve the tissue quality for molecular tests. Herein, we report an electrically powered snap freezing device as an alternative to quenching the vial in liquid nitrogen and therefore can be used directly at the location where the tissue is acquired. This device also facilitates the study of the effect of freezing conditions on the various molecular processes in the samples. Cooling experiments of a vial in the snap freezing device show that the cooling rates similar to or faster than quenching in liquid nitrogen are feasible. We performed experiments with several set point conditions and compared the results with a mathematical model.

## Introduction

Access to frozen biological material is necessary to accelerate the implementation of molecular biology techniques^1–3^. Current procedure is to immerse a vial containing the biological material in liquid nitrogen^4^. Liquid nitrogen is cheap and is available in most hospitals, but requires qualified technicians to operate thereby increasing the cost of the health care. Moreover liquid nitrogen cannot be used in operation theatres due to safety regulations. Therefore the biological material must be transported quickly to a different laboratory where snap freezing is performed^5^.

Changes in molecular profiles of obtained samples may occur once the tissue is removed from the patient, during the pre-fixation time and during sample processing. It is known that collection procedures of tissue can have a significant impact on the quality and the type of diagnostic test which is performed. The time between removal and fixation is known as cold ischemia time and can have significant changes on the gene and protein reactions in a cell^6^. A snap freezing device that do not use liquid nitrogen and is powered by a cryocooler will ease the snap freezing procedure and will improve the quality of frozen tissues as transportation for snap freezing can be completely avoided.

In addition, earlier studies show that the critical cooling rate for a high survival rate of the cells is strongly cell-type dependent^7^. This is attributed to the heterogeneous nucleation in slow freezing and homogeneous nucleation in fast freezing. The effect of freezing conditions on the various molecular processes in the tumour tissue samples is not sufficiently known. The flexibility to set the operating parameters in the freezing process (such as cool down time and temperature) will open up the possibility to study these molecular processes in more detail.

**Figure 1:**
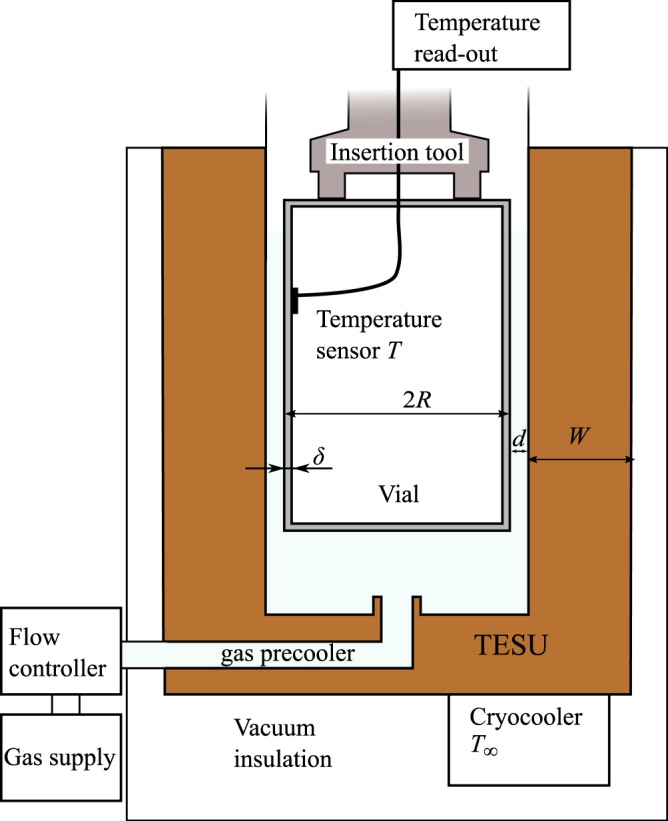
Sketch of the snap freezer.

We built an electrically powered snap freezer with the possibility to adjust the cold sink temperature. The assessment of various cooling principles and the functional design of the snap freezing device is described in our earlier paper^8^. The apparatus has in essence three major components (see Fig. 1): a cryocooler, Thermal Energy Storage Unit (TESU), and a gas handling system. When a vial is inserted into the thermal reservoir, the cooling occurs through a narrow gas-gap between the vial and the reservoir. In this device it is possible to set a constant gas flow through the gas-gap.

Cooling experiments performed with this snap-freezer show that cooling speeds higher than with quenching in liquid nitrogen is achievable. Everyone expects forced flow to substantially increase the cooling speed, but counter intuitively we observed a marginal increase. Narrow gas gaps increase the diffusion of heat from one wall to the other, but adversely influences the convective heat transfer. In this paper we systematically study heat transfer during cooling of the vial for several settings of the snapfreezing device. We propose a theoretical model of the cooling process and verify this model with experimental data.

## System Design

The vial that is used in this study is made of aluminium and has a wall-thickness *δ* of 0.2 mm, an external diameter 2*R* of 14.5 mm, and a height of 25.0 mm. This vial is used at the Amsterdam Medical Center for snap freezing of the biopsied tissue. When the vial is inserted into the snapfreezing device, the gap *d* between the external vial wall and the cold thermal reservoir is 0.4 mm. The thermal reservoir will be referred to as TESU, which stands for Thermal Energy Storage Unit. The TESU is made of copper, having a mass *M* of 1 kg, resulting in a thermal mass (*M*⋅*c*_*cu*_) of 200 JK^−1^ to 300 JK^−1^ in the temperature range of 80 K to 140 K. As a consequence, the reservoir is expected to increase in temperature at most by 2 K for every cooling procedure. The temperature of the TESU can be controlled by a cryocooler.

The design of the snapfreezing device hinges on the fact that a small gap *d* between the vial and the TESU results in a rapid exchange of heat between the two. As a result of the large separation of length scales, the system can be simplified by a one-dimensional model, in which we need to describe the temperature change of the vial wall, the TESU and the gas in the gap.

Let us now first estimate the total energy change ${\rm{\Delta }}E=m{\int }_{{T}_{\infty }}^{{T}_{{\rm{amb}}}}\,c(T){\rm{d}}T$ to cool down the vial from ambient temperature to the desired temperature of the TESU, *T*_∞_. Here *c* is the vial specific heat and *m* the mass of the vial. Since most mass is at the sidewall of the vial, we obtain *m* ≈ *Aδρ*, where *A* = 2*πL* is the area of the vial wall. Balancing Δ*E* with the heat transferred across the gap, we obtain the cooling time scale of the vial: $t \sim (\rho c\delta A{\rm{\Delta }}T)/(A{k}_{{\rm{g}}}/d) \sim {\mathscr{O}}\mathrm{(1)}$ second. Any convective heat transfer in the gap are of the order unity and alter this time scale by one order of magnitude at most. Using *t*, one can estimate the Fourier number ${\mathcal F} o=\alpha t/{\ell }^{2}$, to determine whether or not temporal effects are important in the diffusion equation. Here $\ell$ and *α* are the characteristic length scale and thermal diffusivity of the vial, gas, and TESU, respectively. For the vial, $\ell$ is the wall thickness *δ*, for the gas it is the gap thickness *d* and for the TESU we use the wall thickness *W*. We find that the Fourier number is much larger than unity for all three domains, see Table 1. This implies that the temperature profiles inside the three domains adapt instantly with respect to the cooling time scale of the vial. As a result, the profile in the gap can be obtained by solving the steady *non-linear* heat equation in the gap, where the non-linearity is a consequence of the relative large variation of the thermal conductivity *k*_g_ over the temperature range. One can represent the heat flux using ${\bar{k}}_{{\rm{g}}}=\mathrm{1/}{\rm{\Delta }}T\int \,k(T){\rm{d}}T$ as the *mean* conductivity of the gas to obtain $q^{\prime\prime} =-\,{\bar{k}}_{{\rm{g}}}{\rm{\Delta }}T/{d}$. Here *d* is the gap width and Δ*T* the temperature difference between the vial wall and the TESU wall.

**Table 1:** Estimation of the characteristic lengthscale $\ell$, thermal diffusivity *α* and thermal timescale $\tau ={\ell }^{2}/\alpha$. Values evaluated at 100 K, for a aluminium vial, helium filled gap and copper TESU.

	$\ell$ [m]	*α* [m^2^s^−1^]	*τ* [s]
vial wall *δ*	2 × 10^−4^	1 × 10^−4^	4 × 10^−4^
gap *d*	4 × 10^−4^	2 × 10^−5^	8 × 10^−3^
TESU wall *W*	1 × 10^−2^	1.5 × 10^−4^	6 × 10^−1^

We now estimate the Biot numbers of the vial and the TESU: ${\mathcal B} i=h\ell /k$, using the same characteristic length scales as before. *h* is the heat transfer coefficient, which is for the diffusion across the gap is simply ${\bar{k}}_{{\rm{g}}}/{d}$ and *k* is the thermal conductivity of the vial and TESU, respectively. Evaluation of ${\mathcal B} i$ lets us discard the possibility of temperature gradients occurring inside the vial and the TESU as ${\mathcal B} i\ll 1$ for both cases. As a consequence we can treat the vial and the TESU as a lumped capacitance.

The energy Δ*E* change of the vial during cooling is much smaller than the energy required to heat the TESU significantly, as the latter has a thermal mass (~*Mc*_*cu*_), hence remaining approximately at *T*_∞_ throughout the cooling procedure. The cooling of the vial can therefore be modelled as

1 $${\partial }_{t}T=-\,\varepsilon \frac{{\bar{k}}_{{\rm{g}}}(T){A}_{{\rm{v}}}}{{m}_{{\rm{v}}}c(T)}\frac{T-{T}_{\infty }}{d}$$

The constant *ε* represents a fitting parameter, modelling effects which enhance the heat transfer, which will be discussed later. This equation is solved numerically using *T*(0) = *T*_amb_ = 293 K, the ambient temperature of the lab.

## Results

### Cooling affect by conduction

**Figure 2:**
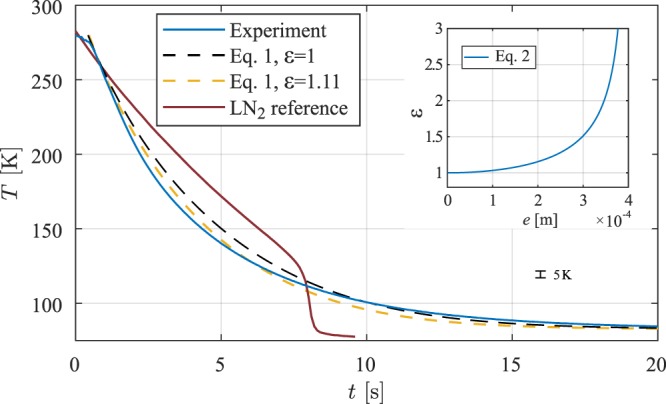
The experimental data is the average of four different measurements, which differed at most 3 k, smaller than the line thickness. The inset shows the enhancement in heat transfer *ε* as a result of a improperly centred vial, described by Eq. 2.

We first perform experiments with the TESU cooled to *T*_∞_ = 80 K to obtain a qualitative comparison to the reference case of quenching the vial in liquid nitrogen, a separate experiment carried out in our lab. Here we submerged the vial, initially at *T* = *T*_amb_ into a bath of liquid nitrogen and recorded the temperature during cooling. The averaged results for four cooling measurements in our snapfreezer are presented in Fig. 2. Aside from the delay of half a second as a result from the vial insertion protocol, we find excellent reproducibility: The largest difference between the four measurements was only 3 K. We now compare the data with the solution of Eq. 1. Both the model and the experimental data cool down much faster for *T* > 120 K than quenching in liquid nitrogen. Though the model already describes the cooling behaviour quite well, better agreement can be achieved when allowing for an amplification of heat flux by a factor *ε*, as can be seen by the yellow curve. With only a slight increase of 11% we obtain much better agreement.

We now explore several possible origins of this enhancement. Firstly, when the vial axis is not concentric to the device axis, an enhancement in heat transfer *S*(*e*) occurs, depending on the eccentricity *e*, which is the displacement of the vial axis from the device centre. For *e* = 0, the vial is placed exactly in the centre, while for *e* = 0.4 mm the vial is touching the wall of the TESU. The enhancement in heat transfer is given by^9^

2 $$\varepsilon =\frac{S(e)}{S\mathrm{(0)}}=\frac{{\cosh }^{-1}(\frac{{R}^{2}+{(R+d)}^{2}}{2R(R+d)})}{{\cosh }^{-1}(\frac{{R}^{2}+{(R+d)}^{2}-{e}^{2}}{2R(R+d)})},$$

where *R* is the vial radius. In the inset of Fig. 2, *ε* is shown for various values of e. We find the best fit to the data using *ε* = 1.11, corresponding with an eccentricity of 0.17 mm. This however is not unreasonable, since the vial is inserted manually.

Secondly, any tilting of the vial inside the TESU makes one side at the top and the opposite side at the bottom of the vial approach closer to the wall. From the geometry we calculated that the maximum tilt was 1.9°, which results in an enhanced heat transfer of *ε* = 1.48 as found by a numerical study of this effect (see the Methods section). Thirdly, we find a faster cooling rate of the vial in our measurements for the first two seconds. This might be a result of the insertion method, where the gas in the TESU is displaced by the vial. As a result of this, the gas flows out of the TESU, enhancing the cooling rate. This effect however decays over time as a result of the viscosity of the gas.

**Figure 3:**
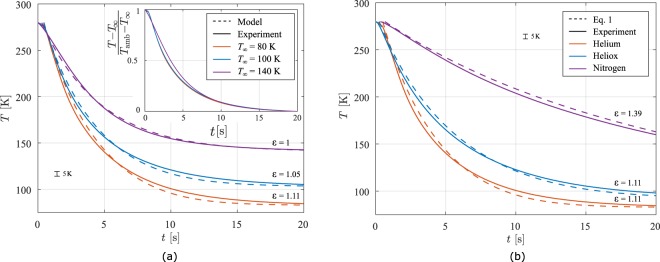
The vial was cooled using different TESU temperatures (**a**) or gas content in the gap (b). Helium gas was used for the experiments of panel (a), whereas in panel (b), the TESU was set to *T*_∞_ = 80 K. The dashed lines show the solutions to Eq. 1, using *ε* as a fitting parameter, see the text. The inset of panel (a) shows that the time scale of the cooling do not differ much when varying the TESU temperature.

As mentioned in the introduction, the systems allows to study the effect of freezing conditions on the quality of the material stored in the vial. Therefore, we test the apparatus for different TESU temperatures *T*_∞_. For every measurement, the vial is taken out of the snap freezer, allowing the vial to equilibrate to the temperature of the lab. Since each experiment involves a new insertion procedure, the positioning is expected to differ for every measurement, leading to a different *ε*. Results of four averaged cooling measurements are presented with the solution of Eq. 1 in Fig. 3a for three different set point temperatures *T*_∞_. It is clearly visible that the cooling rate can be controlled well by the setpoint temperature, since ∂_*t*_*T* ∝ *T* − *T*_∞_. For *T*_∞_ < 140 K we find that the cooling rate is higher in the liquid nitrogen in the temperature range of 300 K to 220 K, so called danger zone where ice crystals may damage the tissue^10^. The cooling rate is well captured by our one-dimensional model. However a higher cooling rate is observed initially, most likely as a consequence of forced convection originating from the insertion method, as described above. This effect has decayed after a few seconds and the predicted cooling rate is within 15% of the experimental value.

The non-exponential cooling characteristic in liquid nitrogen lays in the fact that during cooling the film thickness becomes smaller, while in our apparatus we have a constant gas-gap thickness. The main contribution to the rapid cooling however is that in our experiments we used helium gas, which has a high thermal conductivity compared to nitrogen. It is therefore interesting to compare the performance using different gases in the gap.

Three types of gases are used, namely helium, nitrogen, and heliox. The latter is a mixture of both helium and oxygen in a composition of 79 to 21 mass percent, respectively. Heliox is frequently used in hospitals, which makes it easy to use for the snap freezer^11^. Prior to insertion we flush the insertion volume with the gas, then stop the flow and insert the vial into the snapfreezer. We measure the temperature *T* during cool down, which are displayed in Fig. 3. As expected, the vial in case of nitrogen in the gas-gap cools down much slower compared to the helium and heliox gas as a result of the lower thermal conductivity. A small difference can be found for Heliox, compared to helium, since it contains 21% of oxygen, lowering the conductivity of the gas mixture. The model to determine the thermophysical properties of heliox is described in the Methods section.

### Cooling effect of forced convection

**Figure 4:**
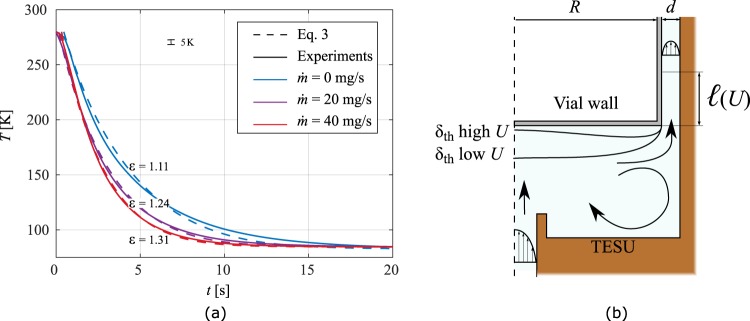
Cooling results (**a**) for vials in the presence of a continuous gas flow jet at various flow rates of helium. As sketched in (**b**), the jet is impacting the bottom of the vial, where the arrows indicate the main flow characteristics.

As mentioned earlier, gas can be injected into the device to flush the system. Where before the flow was stopped before the insertion of the vial, we here study cooling of the vial where the gas is still flowing. The gas is injected from below, impacting the bottom of the vial and then flowing through the gap between the TESU and the side wall of the vial. The results for a flow rate of 20 and 40 mgs^−1^ of helium is shown in Fig. 4a, together with a schematic of the flow in Fig. 4b. It can be seen that the cooling rate is enhanced with increasing flow rate. However, the flow has only a small influence on the cooling of the vial.

By the aid of a commercially available finite elements simulation software^12^ we simulated the effect of the flow on the cooling rate (details can be found in Methods section). Since we consider a quasi-static problem, the divergence of the enthalpy field should be zero. Applying the divergence theorem one can thus evaluate the fluxes at the boundary to investigate how the flow alters the energy fluxes in the system. Simulations were performed with a helium flow up to 40 mgs^−1^, comparable with experimental conditions. The results are listed in Table 2, where the various heat fluxes are calculated, together with the change in enthalpy of the gas Δ*H*, which will be discussed later. We find that the majority of the heat exchange still occurs by diffusion across the thin gap. Only flow rates larger than 20 mgs^−1^ increased the heat flux by more than 10%, see Table 2. Three effects can be identified in the case of forced flow in the gap:

**Table 2:** Simulation results for a vial at *T* = 290 K and the TESU at 80 K, where a helium gas flow at 80 K is forced around the vial.

Mass flow $\dot{{\boldsymbol{m}}}$ [mg s^−1^]	${\dot{{\boldsymbol{Q}}}}_{{\bf{vial}}}$ [W]	${\dot{{\boldsymbol{Q}}}}_{{\bf{TESU}}}$ [W]	${\dot{{\boldsymbol{Q}}}}_{{\bf{outlet}}}$ [W]	${\boldsymbol{\Delta }}{\dot{{\boldsymbol{H}}}}_{{\bf{est}}}$ [W]	$\frac{{\dot{{\boldsymbol{Q}}}}_{{\bf{vial}}}{\boldsymbol{(}}\dot{{\boldsymbol{m}}}{\boldsymbol{)}}}{{\dot{{\boldsymbol{Q}}}}_{{\bf{vial}}}{\boldsymbol{(}}\dot{{\boldsymbol{m}}}{\boldsymbol{=}}{\bf{0}}{\boldsymbol{)}}}$	$\frac{{\dot{{\boldsymbol{Q}}}}_{{\bf{outlet}}}}{{\dot{{\boldsymbol{Q}}}}_{{\bf{vial}}}}$
0	−68.2	68.0	0	0	—	—
4	−71.1	69.1	1.8	2.0	1.04	0.03
20	−80.0	70.1	9.0	10.4	1.17	0.11
40	−88.7	69.0	18.0	20.8	1.30	0.20

${\dot{Q}}_{{\rm{vial}}}$, ${\dot{Q}}_{{\rm{TESU}}}$ and ${\dot{Q}}_{{\rm{outlet}}}$ are the heat flows from the vial, TESU and carried by the flow, respectively. As a reference, evaluation of Eq. 1 for the same temperature difference yields 65.3 W.

Firstly, the length changes for which convection in the gap plays a role. The enhanced heat transfer across the gap is however a minor effect, since it is much smaller than the length of the gap. This convection-length can be estimated using classical duct-flow analysis and yields $\ell  \sim \frac{{d}^{2}U\alpha }{2\cdot {\mathscr{N}}u}$. Here ${\mathscr{N}}u=7.54$ the Nusselt number for parallel plates, well suited since the gap $d\ll R$. For the flows studied we obtain a maximum $\ell \approx 1\,mm$ for $\dot{m}=40\,{{\rm{mgs}}}^{-1}$, much smaller than the length of the vial, being 22 mm. This implies that beyond $\ell$ convection is not important and no enhancement of heat transfer occurs, it is solely conduction from the vial to the TESU.

Secondly, the jet characteristics change significantly for higher flow rates. This causes thinning of the thermal boundary layer at the bottom of the vial, resulting in an enhanced cooling from this area. Nevertheless, the area of the bottom is small compared to the sidewalls of the container, resulting in an increase in cooling rate of 10% for the highest flow rate calculated.

Thirdly, the gas undergoes a change in temperature when flowing past the vial. Initially, the gas is at the temperature of the TESU, as it is cooled by the cryocooler using a heat exchanger. When it flows past the hot vial, the gas heats up, changing in enthalpy. When the gas exits the gap, the temperature profile across the gap is approximately a linear profile. The enthalpy increase with respect to the injection state is ${\rm{\Delta }}\dot{H}=2\pi {\int }_{R}^{R+d}\,{\rho }_{{\rm{g}}}{c}_{{\rm{g}}}u(T-{T}_{\infty }){\rm{d}}x$, where *R* is the radius of the vial and *T* and *u* the temperature and velocity profiles in the gap respectively. This integral can now be approximated in the aforementioned case of a linear temperature profile, using the mass flow $\dot{m}:\,{\rm{\Delta }}{\dot{H}}_{{\rm{est}}}\approx \frac{1}{2}\dot{m}{\bar{c}}_{{\rm{g}}}(T-{T}_{\infty })$, where ${\bar{c}}_{g}$ is the gas specific heat at constant pressure evaluated at the mean temperature in the gap. From Table 2 it is clear that this approximates deviates only by 10%. We therefore can extend the earlier model without flow described by Eq. 1 with our approximation of ${\rm{\Delta }}\dot{H}$:

3 $$mc(T){\partial }_{t}T=-\,\varepsilon {\bar{k}}_{{\rm{g}}}(T)A\frac{T-{T}_{\infty }}{d}-\frac{1}{2}\dot{m}{\bar{c}}_{{\rm{g}}}(T-{T}_{\infty }\mathrm{).}$$

Note that the residence time of the gas in the gap is much smaller compared to the cooling rate of the vial, justifying the assumption that the gas leaves the gap at approximately half the temperature difference between the vial and the TESU. Moreover, the total heat capacity of the gas leaving the gap per unit time is also much smaller than the total heat capacity of the vial.

Based on our measurements and simulations we can conclude that the effect of convection by continuous gas flow is small as a result of the high ratio between the length of the vial and the small gap. Most energy is still transferred in the form of diffusion across the gap, while the heating of the cold gas accounts for at most 20% of increased cooling rate. An indirect enhancement in this diffusion is also noted. We observe in Fig. 4a that *ε* also increases with increasing flow rate. For a purely viscous flow the centre configuration is unstable and any misalignment forced the vial more out of centre. Overall, the usage of gas injection is not the most optimal way to control the cooling rate of the vial, as large quantities of gas are discharged and lost to the environment. The cooling rate can be controlled more easily by adjusting the temperature *T*_∞_ of the TESU.

The mathematical analysis presented in this paper and its validation with experimental data will allow exploration of the parameter space of the snap freezing device so as to design a cost effective apparatus; as an example nitrogen gas with a narrower gas-gap of 0.1 mm will have similar cooing rate as liquid nitrogen. Adapters to accommodate various vial sizes could also be built. As a next step in this development, clinical tests with tissue samples will be performed at the VUmc Cancer Center of Amsterdam to benchmark this device with the liquid nitrogen protocol.

## Discussion

A device to freeze biosamples enclosed in a vial is developed, which is powered by a low capacity cryocooler. Contact gas and the size of the gas-gap between the vial and the TESU influences the cooling speed. A mathematical model is developed to capture the cooling dynamics of the model, which is then verified with the experimental data. The contribution of heat transfer in the gas-gap due to the convection is small compared to the thermal diffusion through the gas-gap therefore, the gas flow through the gas-gap marginally influences the cooling speed of the vial. We have also shown that the mis-alignment of the vial to the device axis results in the increased cooling speed.

## Methods

### Hardware

**Figure 5:**
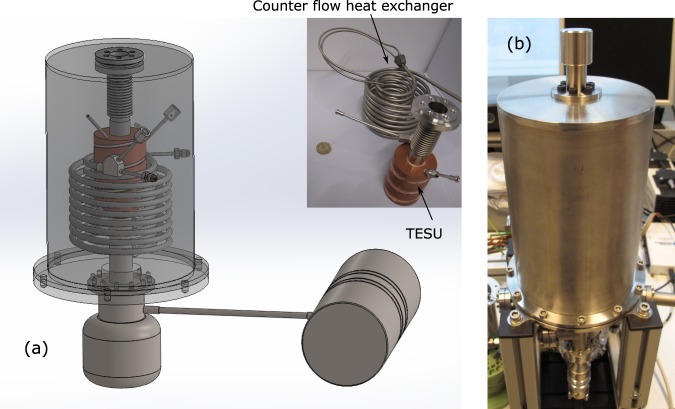
CAD model of the snapfreezer showing the cryocooler and the inner details (**a**). As built assembled apparatus (**b**).

The device with which cooling experiments are performed is shown in Fig. 5. The vial is inserted using a holder from the top opening in the device.

### Numerical Methods

We used a commercial finite elements package^12^ to simulate two aspects of vial positioning in the TESU, as well as the influence of convection. For all these studies, we used the geometry and materials as described in the main body of the paper. The justification of the lumped-capacity model was demonstrated there as well, and hence the simulations were performed by seeking the steady-state solution of the system, where we modeled only the gas domain. The quasi-static cooling of the vial was simulated by varying the boundary condition of the gas domain, while keeping the opposite boundary condition fixed at the TESU temperature *T*_∞_ = 80 K. The advantage of solving the steady state problem is that it is not computationally expensive.

**Figure 6:**
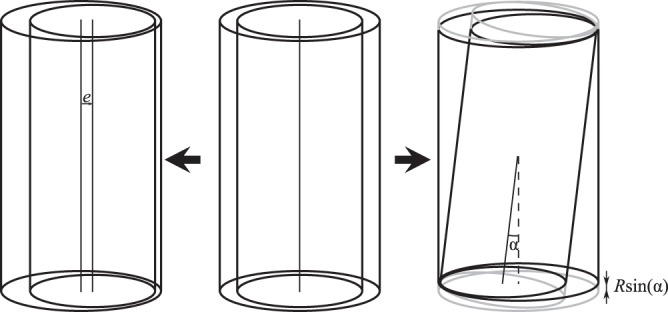
Sketch of a vial being shifted (left) from the center or tilted (right).

In the first two numerical studies we neglected diffusion from the top and bottom of the vial, as the analytical model do not include these as well. The vial is thus modeled as a cylinder, where the vial and the lid are modeled as one piece. From the large difference between the vial radius and the maximum eccentricity, which is the gap size, this effect is indeed negligible. The limit of direct contact was not explored as the diverging heat flux will induce local cooling effects, which is beyond the present scope of the study. As explained in the paper, one can capture any temperature dependency of the thermal conductivity by using an effective thermal conductivity. For simplicity, we used a constant conductivity here, so we only solve $\nabla$^2^*T* = 0. We then evaluate the fluxes on the vial wall ${\int }_{A}\,\overrightarrow{q}\cdot \overrightarrow{n}\,{\rm{d}}A$, where *A* and $\overrightarrow{n}$ are the area and normal of the vial side-wall respectively and $\overrightarrow{q}=-\,{\bar{k}}_{g}\nabla T$ is the local heat flux. Since we are interested in the enhancement in heat transfer and the problem is linear, the exact values for the temperature differences and conductivities are not important here as they cancel after the normalization. A sketch of the shifted or tilted vial can be seen in Fig. 6.

#### Eccentricity

We first validated the enhancement in heat transfer *ε* by an eccentric positioning and found good agreement, see Table 3.

**Table 3:** Numerical validation of Eq. 2, where an enhancement in heat flux occurs as a result of an eccentric position *e* of the vial in the snapfreezer.

Eccentricity *e* [m]	0	1 × 10^−4^	2 × 10^−4^	3 × 10^−4^	3.9 × 10^−4^
*Q*(*e*)/*Q*(0)	1	1.03	1.15	1.51	4.31
Equation 2	1	1.03	1.16	1.51	4.45

Though one side of the vial is moving away from the side wall with increasing *e* the opposite side gets closer, leading to an increase in total heat flux.

#### Affect of tilt

Next, we positioned the vial again in the center (*e* = 0) and tilted it inside the gap. The maximum tilt achieved was 1.9°, in agreement with geometrical calculations. In this position, the top of the vial is touching the wall of the TESU, while on the same side, the bottom is almost at 0.8 mm away from the wall. On the opposite side of the vial, the top is away from the TESU, while the bottom makes contact. The increase in total heat flux is presented in Table 4, where we see that the maximum increase in heat transfer *ε* is 1.48 times the value of zero tilt.

**Table 4:** Calculated enhancement in heat flux as a result of an tilted position of the vial in the snapfreezer.

Tilt angle [°]	0	0.5	1	1.5	1.75	1.9
*Q*/*Q*(0)°	1	1.02	1.06	1.16	1.27	1.48

#### Flow

Let us now consider the case that a helium flow is forced around the vial, as sketched in Fig. 4. The gas flow is injected from the bottom via the tube. The simulation is still steady state, as explained before and we assume an axi-symmetric domain, as the vial is positioned in the axis of symmetry (i.e. no tilt and eccentricity). We solve the flow field, allowing for compressibility effects as a result of the large density differences expected from the large temperature difference between the vial and the TESU wall (which can be direct after insertion 193 K). We thus solve:

4 $$\nabla \cdot (\rho \overrightarrow{u})=0;\,\rho (\overrightarrow{u}\cdot \nabla (\rho \overrightarrow{u}))=-\,\nabla p+\nabla \cdot (\mu (\nabla \overrightarrow{u}+{(\nabla \overrightarrow{u})}^{T})-\frac{2}{3}\mu (\nabla \cdot \overrightarrow{u}){\bf{I}}),$$

where *ρ* and *μ* are the gas density and viscosity respectively and *p* is the pressure field. **I** is the identity matrix and ^*T*^ the transpose operator Eq. 4 is coupled to the heat equation:

5 $$\nabla \cdot (k\nabla T)=0,$$

in which *k* is the thermal conductivity of the gas. All gas properties are evaluated locally, since they depend on the local temperature. The problem is closed using the equation of state, coupling the pressure field, density and temperature, where the ideal gas law was used:

6 $$\rho =pM/(RT),$$

where *M* is the molar mass of the gas and *R* = 8.314 JK^−1^ mol^−1^ the universal gas constant.

The problem is subject to an open boundary at the top ($\nabla$*p* = 0, *p* = 10^5^ Pa) and no slip is applied on all solid boundaries. The vial wall is at 293 K, while the tube and TESU are at 80 K. We assume the injected gas flow to be fully developed and cooled to 80 K. The mass flow is used as a control parameter.

We used a random triangulation of the domain, while rectangular boundary layers were used near the side walls. Additional refinement was imposed at the corners, capturing potential boundary layer separation. Over twelve thousand elements were used, and the solution was tested against coarser meshes to study the convergences of the solution.

The solution was found for various mass flow rates $\dot{m}$, for which the solution is still a laminar flow. The highest flow rate was found to be in the tube of the gas inlet. The heat transfer was evaluated at different positions: (1) the wall of the vial, using ${\int }_{A}\,k\nabla T\cdot \overrightarrow{n}\,{\rm{d}}A$, where *A* is now the vial wall, bottom and top. (2) the change in enthalpy of the gas leaving the gap at the top of the vial: ${\rm{\Delta }}H=2\pi {\int }_{R}^{R+d}\,{\rho }_{{\rm{g}}}{c}_{p,{\rm{g}}}(T-{T}_{\infty })\overrightarrow{u}\cdot \overrightarrow{n}\,{\rm{d}}x$ and (3) heat transferred from the gas into the TESU, evaluated similarly as (1). Evaluation of the heat exchanged at the top of the vial showed no significant contribution.

### Thermal properties of Heliox

Little is documented about the properties of Heliox. We therefore use the model of Mason and Saxena^13^ to describe the thermal conductivity and its temperature dependency, where they extended the empirical model of Wassiljewa^14^. Here, the conductivity of a mixture is given by

7 $${k}_{{\rm{m}}}=\sum _{i=1}^{n}\,\frac{{y}_{i}{k}_{i}}{\sum _{j=1}^{n}\,{y}_{j}{A}_{i,j}}\mathrm{.}$$

*i* and *j* denote the components in the mixture, with their molar fraction *y*. *A*_*i*,*j*_ is a function, yet to be determined. Equation 7 reduces for a two component system to:

8 $${k}_{{\rm{m}}}=\frac{{y}_{{\rm{He}}}\,{k}_{{\rm{He}}}}{{y}_{{\rm{He}}}+{y}_{{\rm{Ox}}}{A}_{\mathrm{1,2}}}+\frac{{y}_{{\rm{Ox}}}\,{k}_{{\rm{Ox}}}}{{y}_{{\rm{He}}}{A}_{\mathrm{2,1}}+{y}_{{\rm{Ox}}}},$$

where the subscripts He and Ox refer to the helium and oxygen components. Here *A*_1,1_ = *A*_2,2_ = 1. The interaction between the helium and oxygen gas however are modelled as follows:

9 $$A\mathrm{(1,2)}=\frac{e{[1+{({k}_{{\rm{He}}}/{k}_{{\rm{Ox}}})}^{\mathrm{1/2}}\cdot {({M}_{{\rm{He}}}/{M}_{{\rm{Ox}}})}^{\mathrm{1/4}}]}^{2}}{{[8(1+{M}_{{\rm{He}}}/{M}_{{\rm{Ox}}})]}^{\mathrm{1/2}}},$$

10 $$A\mathrm{(2,1)}=\frac{e{[1+{({k}_{{\rm{Ox}}}/{k}_{{\rm{He}}})}^{\mathrm{1/2}}\cdot {({M}_{{\rm{Ox}}}/{M}_{{\rm{He}}})}^{\mathrm{1/4}}]}^{2}}{{[8(1+{M}_{{\rm{Ox}}}/{M}_{{\rm{He}}})]}^{\mathrm{1/2}}},$$

in which *M* is the molar mass and *e* = 1. Data for *k*_He_ and *k*_Ox_ was obtained from NIST^15^ for the temperature range of 80 till 300 K. Then, Eq. 7 was evaluated and then fitted to obtain a linear relation *k*_m_ = *a*_m_ + *b*_m_*T*, used in solving Eq. 1. We found *a*_m_ = 33.49 × 10^−3^ Wm^−1^K^−1^ and *b*_m_ = 0.41*× 10*^*−3*^ Wm^−1^K^−2^.

## Data Availability

The datasets generated during and/or analysed during the current study are available from the corresponding author on reasonable request.
